# Elucidating neuroepigenetic mechanisms to inform targeted therapeutics for brain disorders

**DOI:** 10.1016/j.isci.2025.112092

**Published:** 2025-02-22

**Authors:** Benjamin H. Weekley, Newaz I. Ahmed, Ian Maze

**Affiliations:** 1 Nash Family Department of Neuroscience, Friedman Brain Institute, Icahn School of Medicine at Mount Sinai, New York, NY 10029, USA; 2 Department of Pharmacological Sciences, Icahn School of Medicine at Mount Sinai, New York, NY 10029, USA; 3 Howard Hughes Medical Institute, Icahn School of Medicine at Mount Sinai, New York, NY 10029, USA

**Keywords:** Therapeutics, Epigenetics, Neuroscience

## Abstract

The evolving field of neuroepigenetics provides important insights into the molecular foundations of brain function. Novel sequencing technologies have identified patient-specific mutations and gene expression profiles involved in shaping the epigenetic landscape during neurodevelopment and in disease. Traditional methods to investigate the consequences of chromatin-related mutations provide valuable phenotypic insights but often lack information on the biochemical mechanisms underlying these processes. Recent studies, however, are beginning to elucidate how structural and/or functional aspects of histone, DNA, and RNA post-translational modifications affect transcriptional landscapes and neurological phenotypes. Here, we review the identification of epigenetic regulators from genomic studies of brain disease, as well as mechanistic findings that reveal the intricacies of neuronal chromatin regulation. We then discuss how these mechanistic studies serve as a guideline for future neuroepigenetics investigations. We end by proposing a roadmap to future therapies that exploit these findings by coupling them to recent advances in targeted therapeutics.

## Graphical abstract

**Figure:**
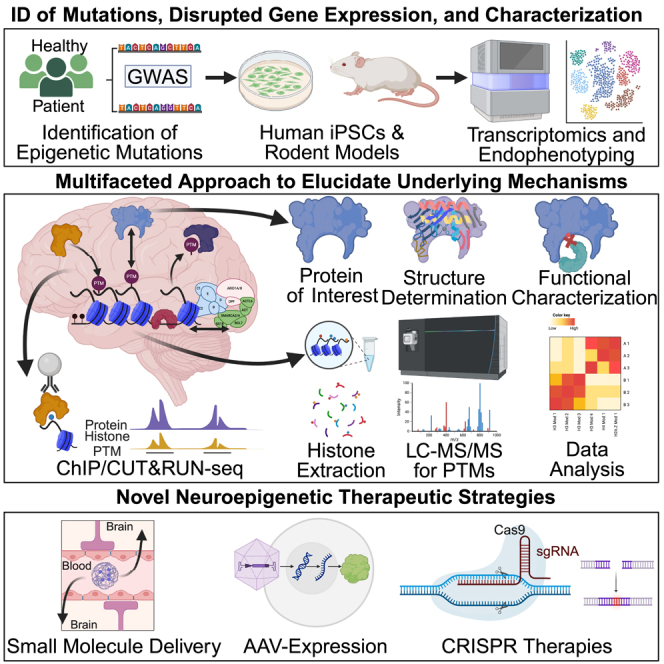

## Introduction

**Figure 1:**
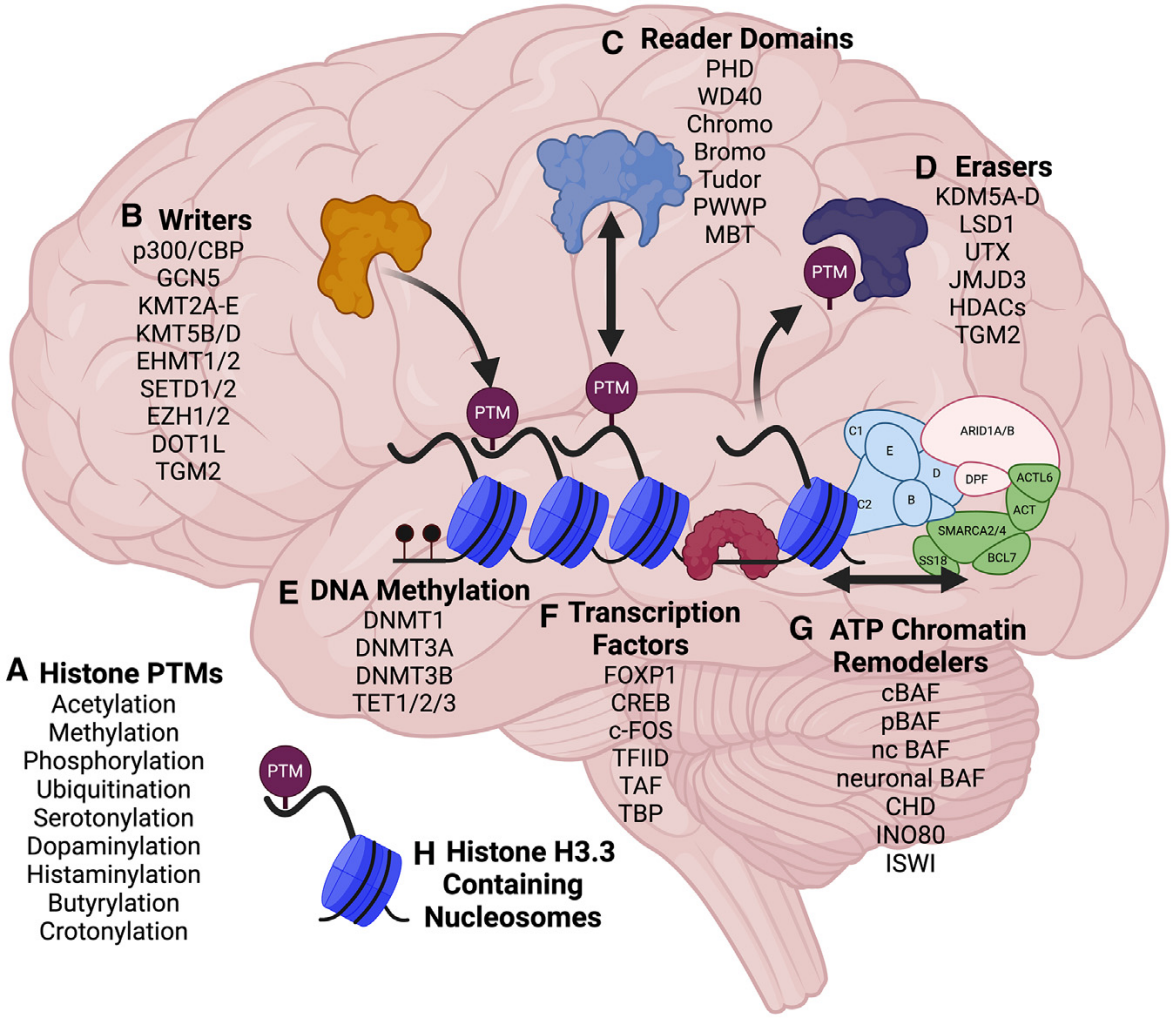
Commonly dysregulated epigenetic modifications, modifying enzymes, “readers,” and remodelers in brain disorders (A) Histone PTMs: select common histone PTMs, as well as recently discovered PTMs, are listed, many of which have been implicated in brain development and disease. The levels of these modifications are controlled by metabolite availability, “writer” and “eraser” expression levels, and the pre-existing epigenetic landscape of histone modifications. (B) Writers: histone PTM “writers” enzymatically modify histones with high specificity with respect to which modifications they deposit and which histones/amino acids they covalently modify. (C) Readers: protein “reader” domains are found within a multitude of proteins, from structural proteins to catalytically active transcriptional machinery. They bind with high affinity to select modifications or combinations of modifications, typically on histone tails, but sometimes within the globular cores of histone proteins. (D) Erasers: histone “erasers” enzymatically remove PTMs from histones with high specificity. An important balance exists within cells and at specific loci between “writers” and “erasers,” which can be influenced by a number of different factors, including chromatin accessibility, expression of the “writers” and “erasers,” and local availability of metabolic donors. (E) DNA Methylation: DNA methylation is controlled by a distinct set of “writers” and “eraser” enzymes, some that are cell replication-dependent and some independent. Levels of DNA methylation can alter transcription factor binding to DNA, as well as downstream transcription. (F) Transcription factors: transcription factors primarily bind to accessible DNA sequences at transcription start sites and enhancers, recruiting transcriptionally active or repressive complexes to facilitate or attenuate transcription. They often have high specificity for specific DNA sequence motifs, and unique interacting partners that can include histone modifiers and ATP chromatin remodelers. (G) ATP chromatin remodelers: adenosine triphosphate (ATP) chromatin remodelers are multi-subunit complexes, which catalytically require ATP to move nucleosomes along the DNA template. This involves nucleosome sliding, exchange, and eviction. Different complexes have been shown to have both overlapping and distinct functions, as well as genomic localizations, due to the varying composition of subunits and interacting partners. Numerous examples exist for cell-type specific chromatin remodeling complexes in both brain and other tissues. (H) Histone H3.3 Containing Nucleosomes: in brain, histone variant H3.3 is increasingly enriched and becomes the dominant H3 isoform throughout neurodevelopment. It is incorporated into chromatin in a replication-independent manner, allowing histone turnover in post-mitotic neural cells. Mutations in one of the two H3.3-encoding genes (*H3F3A/H3F3B*) can severely disrupt brain development and function.

The eukaryotic genome is comprised of double-stranded DNA, which is condensed into the nucleus in the form of chromatin, consisting of ∼147 base pairs of DNA wrapped into nucleosomes, the fundamental repeating units of chromatin fibers and chromosomes in cells.^1^^,^^2^ The nucleosomes themselves are made up of a heterotypic octamer containing four distinct histone proteins—two copies each of H3, H2A, H2B, and H4 (or variants thereof)—which each consist of core globular domains, along with N-terminal and C-terminal unstructured “tails,” which extend from the globular cores and are highly amenable to post-translational modifications (PTMs) (Figure 1A).^3^^,^^4^ Chromatin states are highly dynamic, allowing for changes in DNA-templated processes, such as transcription into messenger RNA (mRNA) for subsequent protein translation. Histone PTMs are added from diverse metabolic donors to induce biochemical states of acetylation, methylation, phosphorylation, monoaminylations, and many more, which can function either *in cis* to alter electrostatic properties of histone-DNA interactions or *in trans* through the recruitment of secondary effector proteins.^5^^,^^6^ Histones interact with a multitude of enzymes, which covalently add PTMs using chemical donors (*writers*), interacting proteins that contain specialized domains that bind to histone PTMs (*readers*), and enzymes that can remove these modifications (*erasers*) (Figures 1B–1D). These intricate processes play a fundamental role in facilitating the recruitment and binding of both transcriptional activating and repressive complexes that can ultimately lead to local changes in gene expression.^7^ Additionally, DNA base pairs can be directly modified through the addition of methyl groups (or their oxidized derivatives; e.g., hydroxymethylation), which can, in turn, alter the binding of transcription factors (TFs) and other components of transcriptional complexes (Figure 1E).^8^ These TFs, which dictate the transcriptional outputs of cells, contain DNA binding domains that have high levels of affinity for specific DNA motifs and are often expressed in a cell-type specific manner (Figure 1F).^9^ Chromatin can also be re-structured by ATP-dependent chromatin remodeling complexes, such as the mammalian SWI/SNF complex, which further aid in facilitating the movement of nucleosomes along the DNA template to allow for (or restrict) TF and/or chromatin modifying protein interactions with histones or DNA (Figure 1G).^10^ mRNAs can also undergo post-transcriptional modifications, such as N6-Methyladenosine (m6A), which are thought to regulate mRNA transport and/or stability, and are controlled by mRNA-specific *writers* and *erasers*.^11^^,^^12^ These epigenetic modifications and their resulting mechanisms have been the focus of extensive study since the discovery of DNA and histone PTMs across many cell-types, as well as in the context of a plethora of human diseases, particularly cancer.

Only more recently (within the last 15–20 years) have epigenetic modifications and their regulation in brain become a prominent focus of research. The average human brain is estimated to have ∼86 billion neurons and ∼85 billion supporting cells (largely glia; e.g., astrocytes, microglia, oligodendrocytes), with many distinct cell-types and/or subtypes present for each.^13^ Given the post-mitotic nature of neurons, neural plasticity (i.e., the capacity of the nervous system to modify itself structurally and/or functionally in response to environmental exposures) is largely facilitated through the integration of both intrinsic and extrinsic stimuli, which ultimately promote adaptations in neuronal wiring and firing, both during neurodevelopment and in adulthood. One mechanism through which the various cell-types in brain accomplish this remarkable plasticity is through epigenetic regulation of both neurons and glia, thereby directly facilitating lasting changes in cell-type specific transcriptional profiles.^14^ These transcriptional changes can impact various functional outputs, such as changes in the expression of receptors, metabolic co-factors, neurotransmitter synthesizing enzymes, and much more. The neuroepigenetics field has now characterized a multitude of these changes, with genome-wide association studies (GWAS) and single gene knock-out (KO) investigations identifying important TFs and chromatin modifiers that are critical for proper neuronal wiring and function in a variety of different brain structures.

Over the past decade, with monumental advancements in DNA and RNA sequencing (RNA-seq) methodologies, researchers have generated considerable data on neural genomes and transcriptional profiles in the context of brain development, disease and responsivity to external stimuli.^15^^,^^16^ These unbiased genomics studies have provided a wealth of phenomenological data for future investigations. While beneficial, much of this work requires additional detailed mechanistic interrogation, insights that will be necessary to faithfully translate these findings into eventual therapeutic outcomes. Excitingly, a select number of more recent studies have begun to mechanistically characterize, particularly from a biochemical perspective, the complex epigenetic and transcriptional changes in brain that underlie how chromatin structure and function contribute to neural plasticity and disease. Such mechanistic interrogations provide more tangible targeting strategies for treating patients and provide a roadmap for subsequent investigations following up on large-scale GWAS and RNA-sequencing studies. In this review, we highlight the methods to identify risk genes, how to test these genes using model systems, the methods to interrogate biochemical mechanisms, and the importance and limitations of integrating genomics into mechanistic neuroepigenetics studies, with the ultimate goal of providing viable avenues of research for effectively bridging these two distinct approaches. Furthermore, we provide viable avenues of research for effectively bridging these two distinct approaches and emphasize potential therapeutic methodologies that could be utilized to target biological processes/pathways in brain that are identified by epigenomics approaches.

## Identification and early characterizations of neurobiological risk genes

### Identification of neurobiological risk genes through advancements in genomic sequencing

**Figure 2:**
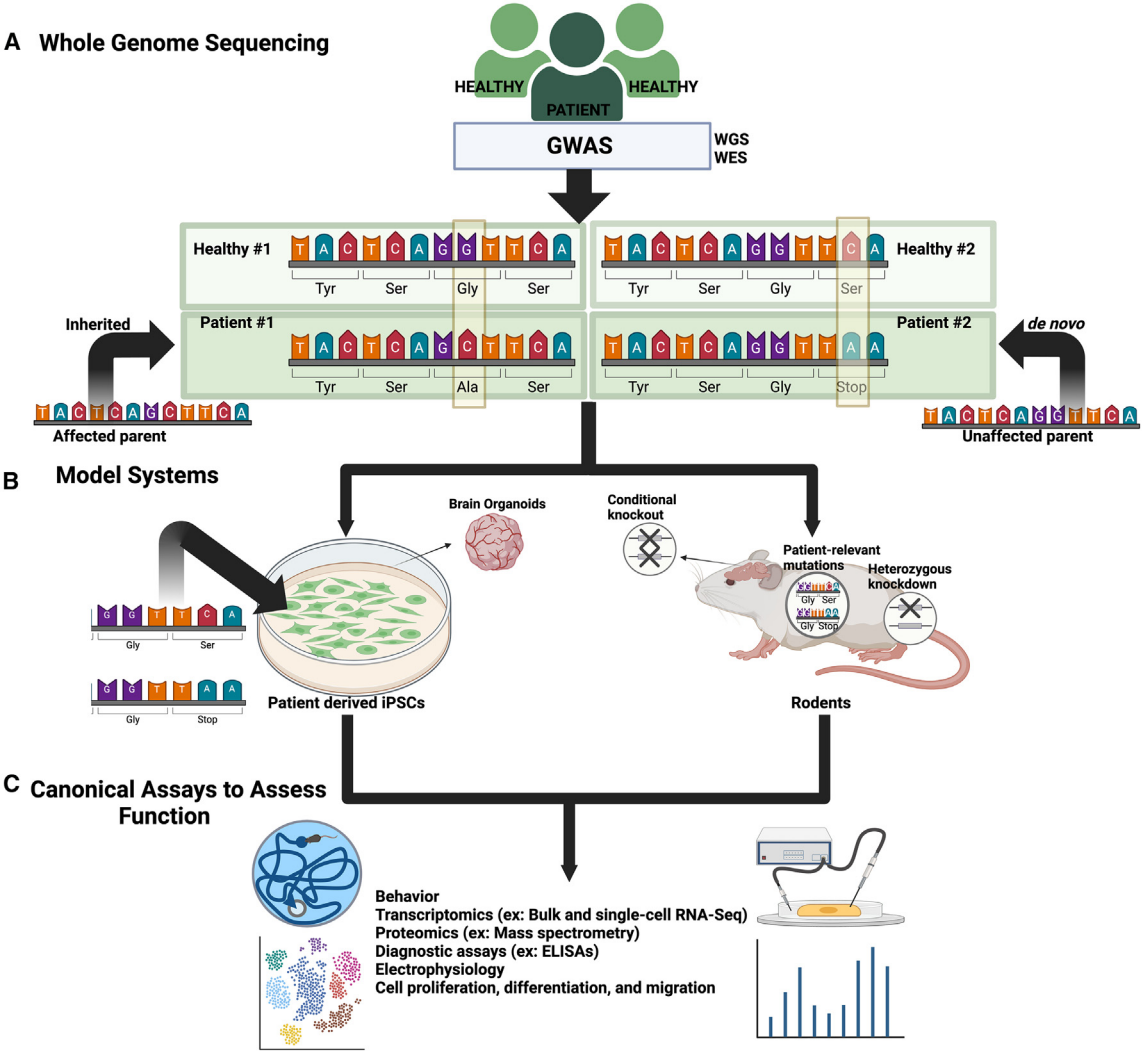
Identification and characterization of genetic variants involved in brain disorders (A) Genome Wide Association Studies (GWAS): GWAS can be carried out using either whole-genome sequencing or whole-exome sequencing of healthy individuals vs. patients with a given brain disorder. The resulting DNA sequencing can be analyzed to identify single nucleotide polymorphisms (SNPs) that associate with a given phenotype. (B) Generation of transgenic model systems for the phenotypic characterization of disease associated mutations: using molecular methodologies, patient-derived induced pluripotent stem cells (iPSCs) can be generated if a given mutation is found in all cell-types, or iPSCs can be genetically manipulated to harbor a specific mutation of interest. Brain organoids can also be derived from iPSCs for further manipulation. Transgenic rodent models can also be generated to knockout a gene of interest (heterozygous vs. homozygous, either globally or conditionally in select cell-types) or knock in a specific mutation, where the gene’s expression can be driven by a specific promoter that is tissue and/or cell-type specific. (C) Downstream assays to assess functional consequences of patient mutations: a number of behavioral, molecular, and biochemical assays can be performed using genetically engineered iPSCs or transgenic rodent models. These include assays such as bulk/single-cell RNA-seq, mass spectrometry, ELISAs, and many more. Characterizing the consequences of patient associated mutations and the mechanisms through which they affect phenotypic outcomes can lead to development of novel targeted therapeutic approaches.

Early identification of brain disease-relevant mutations often relied on sequencing a modest cohort of patients with a specific, well-defined disorder to identify the responsible gene(s). A successful example of this approach was the identification of methyl-CpG binding protein 2 (*MECP2*; a binder of methylated DNA) mutations in Rett syndrome, a well-studied neurodevelopmental disorder (NDD). These methods of gene variant identification utilized approaches such as exclusion mapping and linkage analysis to narrow down specific regions of chromosomes where a mutation may exist.^17^^,^^18^ In the case of *MECP2*, this *a priori* information allowed researchers to screen DNA from Rett syndrome patients and determine the causal gene.^19^^,^^20^ While valuable, these methodologies are less effective in determining the genetic causes of polygenic or highly variable diseases. However, more recent advances in sequencing technologies have now led to incredible breakthroughs in the identification of disease associated genomic variants in larger patient populations. Methods such as GWAS were developed following advancements in DNA-based sequencing technologies, which allow for sequencing of the entire genome rapidly and at continuously decreasing cost. By sequencing individuals’ entire genomes and pairing these findings with phenotypic data, researchers are able to compare large patient cohorts against healthy controls and detect inherited gene mutations, single nucleotide polymorphisms (SNPs), copy number variants (CNVs), and other genome reorganization events, such as insertions and deletions. These methods have proved pivotal toward revealing sequence variations within the human genome, including hundreds of *de novo* and inherited mutations across NDDs^21^^,^^22^^,^^23^^,^^24^^,^^25^ (Figure 2A). Thus, associations can now more easily be made between genotypic variants and the phenotypes observed within a given patient population; as such, GWAS are often conducted by large consortia.^26^^,^^27^^,^^28^^,^^29^ Thanks to these consortia and other efforts, thousands of GWAS analyses have now been performed, providing valuable information across various disease states. GWAS was traditionally conducted through use of microarrays to target pre-determined common variants, but advances in next-generation sequencing are now being employed to identify greater numbers of rare variants. Additionally, techniques such as whole-exome sequencing (WES), which identifies genomic variants within the protein-coding regions of genes, or whole-genome sequencing (WGS), which factors in variants that are found within other regions of the genome (e.g., introns or enhancer regions) (Figure 2A), can also be used to identify neurobiological risk genes.^30^ While WES and WGS are relatively new approaches, they have already proven useful in associating hundreds of rare variants to specific disease states. While the aforementioned DNA sequencing technologies typically rely on short reads (∼100–300 base pairs per read), newer methodologies such as long-read sequencing (LRS) are now being used to reliably sequence tens of thousands of base pairs per read, providing greater resolution of the genome, particularly throughout repetitive and GC-rich regions, and in the context of alternatively spliced transcripts.^31^^,^^32^^,^^33^

While DNA-based sequencing methodologies have greatly enriched our understanding of risk genes/variants genome-wide, more dynamic events, such as the state of the transcriptome, are better understood using RNA-based sequencing technologies. Advancements in RNA-seq have not only allowed researchers to better understand the transcriptome, but have also allowed for the identification of alternatively spliced genes and the identification of different populations of RNA, such as microRNAs and long non-coding RNAs.^34^^,^^35^ While these approaches have long been performed in bulk tissues, recent technological advances have allowed researchers to dive deeper into tissue and cell-type specific risk genes through use of single-cell (sc) and single-nuclei (sn) RNA-seq and ATAC-seq (assay for transposase accessible chromatin sequencing).^36^^,^^37^ The development of these methodologies have provided profound insights into the cell-type specific changes in gene expression and chromatin accessibility that result as a consequence of both adaptive and maladaptive environmental exposures reviewed here in the studies by Zheng et al., Kulkarni et al., and Jovic et al.^37^^,^^38^^,^^39^ Analyses of bulk tissues can obfuscate subtle changes in transcription/chromatin accessibility that may play important roles in disease, yet by focusing on cell-type specific alterations, novel risk genes can be identified at an increasingly rapid pace. These studies can be conducted in tissues collected from patients’ post-surgery or from postmortem brain samples, allowing for investigations of specific brain regions of interest. RNA-based sequencing technologies have also been utilized in spatial transcriptomics, which provides information about the location of gene expression in a cell/tissue, giving a more complete picture of how the transcriptome is changing in different genetic or environmental contexts.^40^^,^^41^ While these technologies remain relatively new, many studies have already begun to utilize them to identify novel risk genes across various brain disorders.^42^^,^^43^

### Epigenetics-related risk genes have been identified in multiple brain disorders

The advanced sequencing methodologies described above have been implemented to identify risk genes—many of which play fundamental roles in chromatin regulation—across a wide range of neurodevelopmental, psychiatric, neurodegenerative and substance use disorders. These include studies identifying risk genes in autism spectrum disorder (ASD),^44^^,^^45^^,^^46^^,^^47^ attention deficit hyperactivity disorder (ADHD),^48^ schizophrenia,^42^^,^^49^^,^^50^ amyotrophic lateral sclerosis (ALS),^51^^,^^52^ Alzheimer’s disease (AD) and Parkinson’s disease^53^^,^^54^^,^^55^^,^^56^^,^^57^^,^^58^^,^^59^^,^^60^ and various substance abuse disorders (SUDs)^61^^,^^62^^,^^63^^,^^64^^,^^65^^,^^66^^,^^67^^,^^68^^,^^69^^,^^70^^,^^71^^,^^72^; the plethora of epigenetics-related genes identified by these studies are highlighted in Table 1. Interestingly, many of these genes identified in these studies overlap across distinct disease states, underscoring their critical roles in maintaining normal brain cell function. Thus, it is clearly important to not only identify genes mutated in specific disease states, but also to understand the nature of these mutations, as they may differentially influence phenotypic outcomes. This collection of studies utilizing WGS, WES, snRNA-seq, and other sequencing methods have provided researchers, caregivers, and families with valuable information as they seek potential treatments.

**Table 1:** Epigenetic regulators linked to neurological disorders identified by sequencing studies

Protein	Gene	Other Common Aliases	Associated Disease(s)	Function
ARID1A	*ARID1A*		Coffin-Siris Syndrome^73^	Component of SWI/SNF complex
ARID1B	*ARID1B*		ASD,^44^^,^^45^ Coffin-Siris Syndrome^73^	Component of SWI/SNF complex
ASH1L	*ASH1L*	KMT2H	ASD^44^^,^^45^^,^^46^	Histone Lysine Methyltransferase
AUTS2	*AUTS2*		ASD,^45^ Rubinstein-Taybi Syndrome^74^	Component of SWI/SNF complex
BRWD1	*BRWD1*		ASD^46^	Component of SWI/SNF complex
CHD2	*CHD2*		ASD^44^^,^^45^	Chromatin remodeler
CHD8	*CHD8*		ADHD,^48^ ASD,^44^^,^^47^^,^^72^ Schizophrenia^49^	Chromatin remodeler
CHD9	*CHD9*		Parkinson’s^57^	Chromatin remodeler
CREBBP	*CREBBP*	CBP, KAT3A	Rubinstein-Taybi Syndrome^75^^,^^76^	Histone acetyltransferase
CREST	*SS18L1*	SMARCL2	Amytrophic Lateral Sclerosis^51^	Component of SWI/SNF complex
DNMT3A	*DNMT3A*		ASD^44^	DNA methyltransferase
DNMT3B	*DNMT3B*		Nicotine dependence^65^	DNA methyltransferase
EHMT1	*EHMT1*	KMT1D	Kleefstra Syndrome,^77^^,^^78^ Schizophrenia^79^	Lysine methylase
EP300	*EP300*	p300	Rubinstein-Taybi Syndrome^75^	Histone acetyltransferase
EZH2	*EZH2*	KMT6A	Kabuki Syndrome^80^	Lysine methylase
HIST1H2BD	*HIST1H2BD*		Cocaine dependence^63^	Histone
Histone H3.3 (G34 mutations)	*H3F3A, H3F3B*		ASD^81^	Histone
JARID1B	*KDM5B*	KDM5B	ASD^44^^,^^45^	Lysine demethylase
KDM4A	*KDM4A*		ADHD^82^	Lysine demethylase
KDM5A	*KDM5A*	JARID1A	ASD^83^	Lysine demethylase
KDM5C	*KDM5C*	JARID1C	X-linked intellectual disability^84^	Lysine demethylase
KDM6B	*KDM6B*		ASD^44^^,^^45^	Lysine demethylase
KMT2E	*KMT2E*	MLL5	ASD^44^^,^^85^	Lysine methylase
KMT5B	*KMT5B*		ASD^44^	Lysine methylase
MBD5	*MBD5*		ASD,^44^ Kleefstra Syndrome^78^	Methyl binding protein
MECP2	*MECP2*		X-linked intellectual disability^20^	Methyl binding protein
MLL1	*KMT2A*	KMT2A, ALL-1, HRX	ASD^45^	Lysine methylase
MLL3	*KMT2C*		ASD,^44^^,^^45^^,^^46^ Kleefstra Syndrome^77^^,^^78^	Lysine methylase
MLL4	*KMT2D*	MLL2	Kabuki Syndrome^86^	Lysine methylase
POFZ	*POGZ*		ASD^44^^,^^45^^,^^46^	Chromatin remodeler
SETD1A	*SETD1A*	KMT2F	Parkinson’s,^57^ Schizophrenia^87^	Histone Lysine Methyltransferase
SETD5	*SETD5*		ASD^44^	Histone Lysine Methyltransferase
SMARCA2	*SMARCA2*		Nicolaides-Baraitser Syndrome,^88^ Schizophrenia^50^	Component of SWI/SNF complex
SMARCA4	*SMARCA4*	BAF190, BAF190A	Coffin-Siris Syndrome^73^	Component of SWI/SNF complex
SMARCB1	*SMARCB1*	BAF47	Coffin-Siris Syndrome,^73^ Kleefstra Syndrome^77^^,^^78^	Component of SWI/SNF complex
SMARCC2	*SMARCC2*	BAF170	ASD^44^^,^^45^	Component of SWI/SNF complex
SMARCE1	*SMARCE1*	BAF57	Coffin-Siris Syndrome^73^	Component of SWI/SNF complex
TLK2	*TLK2*		ASD^44^	Kinase
WAC	*WAC*		ASD^44^	Protein interaction mediator
ZMYND8	*ZMYND8*		ASD^44^	Kinase receptor

A selection of the genes identified by sequencing studies relevant to this review alongside the disease(s) they have been implicated in.

Variants identified in TFs have offered additional insights into the potential epigenetic alterations that contribute to brain development. Mutations in TFs can affect the expression of hundreds of downstream targets (either directly or indirectly through regulation of intermediary transcripts), including genes that affect chromatin regulation and epigenetic mechanisms. Accordingly, variants in TFs are among the class of loci most severely impacting susceptibility to disease.^89^^,^^90^ Such findings have been observed in many GWAS analyses, with dozens of TFs now implicated in neurodevelopmental and neurodegenerative disorders, among others^91^^,^^92^^,^^93^^,^^94^^,^^95^; for example, TFs are among the highest implicated functional groups of all ASD risk genes.^44^^,^^45^^,^^47^ While TFs implicated in disease risk are not discussed in depth here (see in the study by Santos-Terra et al., and Zug et al. ^96^^,^^97^ for more in depth reviews of TF involvement in NDDs), this layer of epigenetic regulation certainly plays an important role in brain development and disease.

In addition, variants of multiple genes encoding for chromatin remodelers and histone modifiers have been identified by these genomics studies (Table 1). For example, many components of the mammalian SWI/SNF complex have now been elucidated in various NDDs, with variants in *SMARCA2*, *SMARCA4*, *SMARCB1*, *SMARCE1*, *ARID1A*, and *ARID1B* having been shown to contribute to a variety of NDDs, such as: Nicolaides-Baraitser syndrome,^88^^,^^98^ Kleefstra syndrome,^77^^,^^78^ Coffin-Siris syndrome,^73^^,^^99^ ASD,^44^^,^^45^ and schizophrenia,^50^ and with ALS,^51^^,^^52^ among others. Studies demonstrating the importance of these subunits in controlling chromatin remodeling and transcription, as well as detailed investigations of the phenotypic effects of these mutations, are discussed below (see mutations in ATP-dependent chromatin remodeler subunits are drivers of brain disorders). Another important chromatin remodeler implicated in ASD, ADHD, and schizophrenia is the chromatin helicase DNA binding protein 8 (*CHD8*), which plays fundamental roles in packaging DNA into chromosomes.^44^^,^^45^^,^^47^^,^^48^^,^^49^^,^^100^^,^^101^ Disruption of this DNA packaging disrupts gene regulation, contributing to the disease phenotypes observed. Of note, related genes, such as: (1) *CHD9* has been implicated by GWAS in Parkinson’s disease^57^; (2) *CHD1* and *CHD2* contribute to ASD/NDDs^44^^,^^45^^,^^102^; and (3) *CHD7* has been implicated in the NDD disorder referred to as CHARGE syndrome.^103^ A multitude of additional histone modifiers—namely, various lysine (de)methylases and acetyltransferases, which we do not discuss in detail here but are reviewed elsewhere^104^—have been identified in these genomics studies and are listed in Table 1.^44^^,^^46^^,^^57^^,^^72^^,^^77^^,^^75^^,^^79^^,^^80^^,^^83^^,^^84^^,^^85^^,^^86^^,^^87^^,^^105^ Interestingly, many of these histone regulators are also implicated in the disorders discussed previously, indicating that multiple aspects of chromatin regulation may be affected within a singular disorder.

Finally, in addition to the variants in TFs, chromatin remodelers and histone modifiers described previously, numerous studies have now identified mutations in the genes encoding the histones themselves. For example, while the canonical, replication-dependent H3 histones, H3.1 and H3.2, are encoded by numerous gene clusters throughout the mammalian genome and are not commonly linked to severe physiological defects (with the exception of specific mutations identified in human cancers),^106^ the replication-independent H3.3 variant is encoded by only two genes (*H3F3A/H3F3B*) and is thus more vulnerable to consequential mutations. This is particularly true in brain where H3.3 exists as the dominant isoform (Figure 1F).^107^ Exome sequencing efforts have identified numerous *H3F3A/H3F3B* germline mutations in individuals with NDDs, including ASD, with these mutations ranging in location from the N-terminal tail of H3.3 to the globular histone core.^81^^,^^108^ Additionally, variants in the histone H2B encoding *HIST1H2BD* gene have been implicated in cocaine addiction, indicating that mutations in histones may be vulnerable to experience-dependent disorders of the nervous system.^63^ Ultimately, such mutations can disrupt histone PTMs, nucleosome stability and *reader* protein interactions, although more detailed mechanistic investigations are needed to elucidate these possibilities.

### Use of rodent and in cellulo models to phenotypically investigate epigenetics-related risk genes

Following identification of risk variants/genes, the next step is often to measure the physiological impact of mutations (e.g., loss-of-function/LoF or missense) in animal or cellular models. For example, in addition to the generation of whole-body or conditional cell-type-specific genetic knockout or mutant animals (e.g., rodents), human induced pluripotent stem cells (iPSCs) harboring missense or LoF mutations can be utilized to comprehensively explore the phenotypic consequences of risk loci (Figure 2B). These models can provide important information regarding the functionality of risk genes in the context of behavior, neural physiology, neuronal morphology, biochemistry, proteomics and/or transcriptional alterations that occur downstream of disrupted loci (Figure 2C). In this section, we will focus on the commonly mutated ASD risk gene *CHD8* as a prime example of the scope of complementary work that can be performed in animal vs. human cellular models to elucidate functions for an epigenetics-related risk gene in the context of disease.

Consistent with predictions from human genomics studies, behavioral analyses in Chd8 haploinsufficient mice have revealed ASD-like phenotypes, with electrophysiological experiments identifying alterations in both excitatory and inhibitory signaling.^101^^,^^109^^,^^110^^,^^111^^,^^112^ These *in vivo* studies also revealed direct interactions between Chd8 and the transcriptional repressor REST, which is thought to play a causative role in disease pathology.^109^ Furthermore, work in mouse models has identified that Chd8, which is highly expressed in oligodendrocytes, plays a crucial role in establishing chromatin landscapes necessary for proper differentiation and myelination by its recruitment of the MLL complex and its subsequent regulation of H3K4me3 deposition.^113^^,^^114^ Importantly, based on these findings, potential treatment strategies can now be tested in these rodent model systems in future studies.

While powerful, rodent models do present with some limitations, such as inconsistent results between studies from different groups. These inconsistencies may be driven by genetic differences between mouse strains, as well as potential sex differences, which are often not explored in great detail. Furthermore, knockout studies may also overlook important phenotypic differences that are induced by patient relevant missense mutations that do not result in complete LoF. For example, contradictory results have been reported with regards to brain size in *Chd8* studies in mice, with heterozygous knockout of the gene having been found to increase brain size, while *in utero* knock-down was observed to impair corticogenesis in an independent study.^101^^,^^109^^,^^115^ Another investigation that compared behavioral and morphological differences in over 30 different strains of mice found that *Chd8* haploinsufficiency resulted in variable results between strains with respect to measures of brain and body weights, as well as cognitive, motor, social, and anxiety-related behaviors.^116^ Testing the impact of a patient-derived CHD8 mutation (N2373K) in mice revealed altered pup-isolation-induced ultrasonic vocalizations and increased self-grooming in males only, while both synaptic and transcriptomic alterations were observed in both sexes, with noticeable differences identified between males and females.^117^ In contrast, a different patient-derived mutation, CHD8 S62X, resulted in repetitive and anxiety-related behaviors in juvenile males (but not in females) and in adults (both sexes).^118^ As well as the importance of taking in interactions with genetic background (sex, strain, etc.) or developmental stage that may contribute to differential phenotypic outcomes, these examples highlight the importance of investigating different patient-derived mutations found within a given gene.

To address some of these limitations of rodent models, *in cellulo* models can be used to model disease phenotypes in a more human-relevant context. This is often done through the use of human stem cell lines (e.g., iPSCs), which allow for the introduction of patient-derived mutations prior to differentiation. These cells can be differentiated into neurons, other neural cell-types, or even organoids using established protocols (Figure 2B). Such approaches also offer a unique opportunity to study non-coding variants located within enhancers or other non-coding regions of the genome, as these mutations cannot typically be easily assessed in animal models. Returning to the example of CHD8, a recent study found that introducing LoF mutations in iPSCs prior to cortical neuron differentiation resulted in decreased neuronal firing and synaptic activity, as well as decreased chromatin compaction across the genome. Additionally, overexpression of wildtype *CHD8* was found to reverse some of the electrophysiological impairments observed.^119^ Another study on CHD8 in human neural progenitor cells (NPCs) found that suppressing *CHD8* reduces H3K36me3 at select genes, which affected splicing patterns^113^ that may contribute to disease relevant phenotypes.

Studies using stem cell-derived neuronal lines, however, also have limitations, since they tend to model only early aspects of neural differentiation/maturation. While these models cannot fully recapitulate the intact neural circuits that allow for behavior in humans or animal models, they can provide important mechanistic insights that researchers can use to develop personalized therapeutics. For example, the more recent generation of brain organoid systems from iPSCs has rapidly evolved, allowing for deeper mechanistic investigations of disease-relevant mutations in circuited neural systems, which can then serve as a valuable tool for screening treatment options, particularly with respect to small molecules and/or CRISPR-based genetic targeting (Figure 2B).^120^^,^^121^ *CHD8* haploinsufficiency in human cerebral organoids has allowed researchers to identify differential trajectories of excitatory vs. inhibitory neuronal development, which may serve as an important foundation for novel therapeutic targeting strategies in patients. Specifically, in early development, *CHD8* haploinsufficient inhibitory neurons display accelerated maturation, whereas in later development, *CHD8* deficient excitatory neurons display an aberrant increase in the numbers of cells, mirroring macrocephaly phenotypes observed in patients.^122^ By studying this mutation in cerebral organoids, both developmental and morphological data were able to be ascertained, highlighting the benefit of this model system. Notably, this, and other organoid studies investigating *CHD8*, utilized scRNA-seq, demonstrating how novel techniques can be merged together to provide a more thorough understanding of patient mutations.^122^^,^^123^ Overall, these various model systems provide valuable information and allow researchers to study how overarching chromatin regulatory processes contribute to human disease; however, differences observed in these various model systems clearly vary depending on the exact mutations introduced, the sex of the animals/cells, and genetic background. As such, mechanistic roles for epigenetics-related risk genes (or their specific mutations in patient populations) cannot be fully elucidated from any one model, making it difficult to define precise, or even global, targeting strategies to treat disease. Thus, we will next discuss how complementary chromatin biochemical approaches may be used to further tease apart mechanisms of action for epigenetics risk genes, which have been identified in targeted and large-scale sequencing efforts.

## Biochemical and mechanistic characterizations of epigenetic modifications and modifiers in brain

### Neuroepigenetics studies are often constrained by low input and high complexity

**Figure 3:**
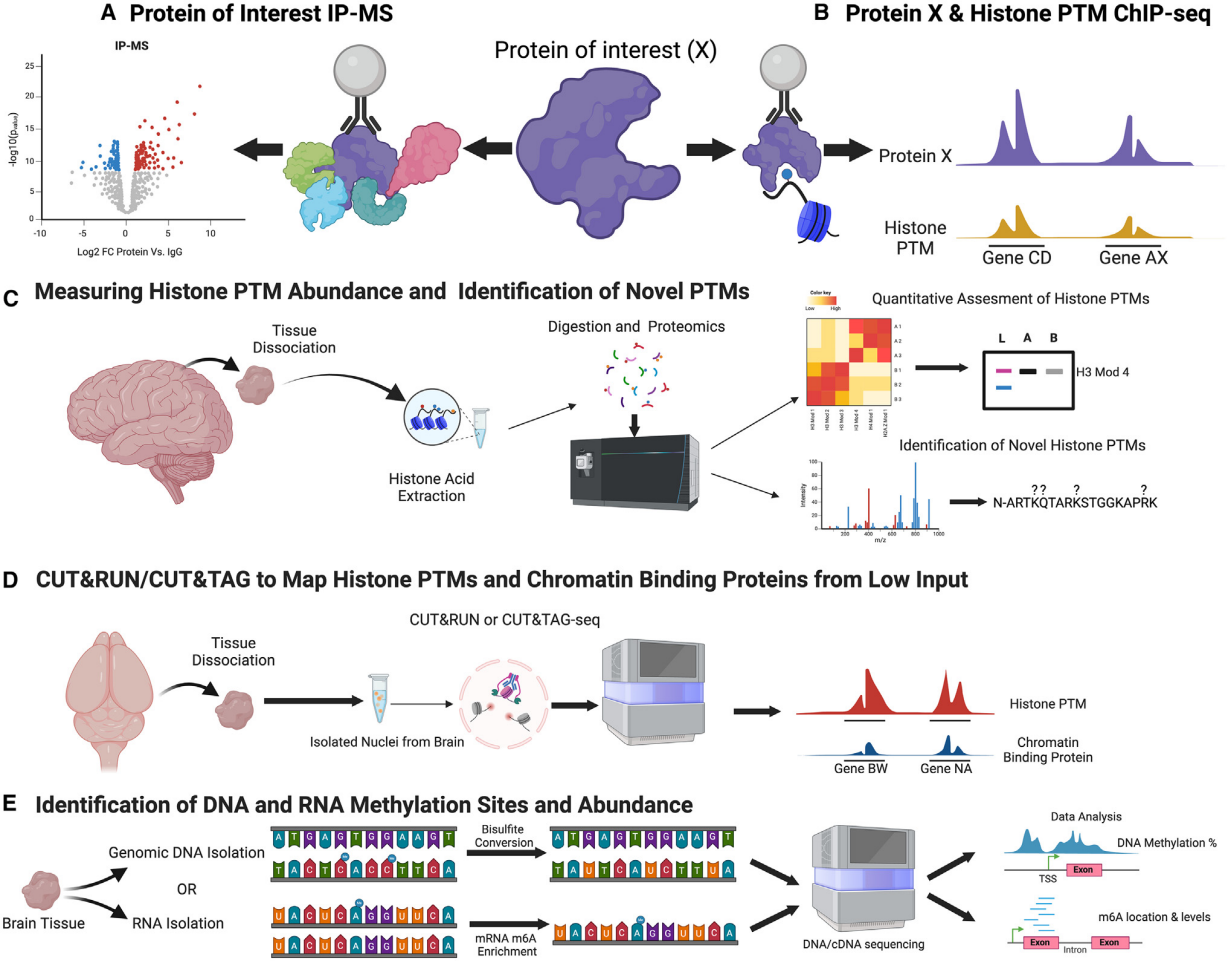
Methods to characterize epigenetic modifications and modifiers in brain (A) Protein immunoprecipitation-mass spectrometry: Antibodies targeting proteins of interest (or a tag, such as FLAG) can be used to immunoprecipitate proteins and any interacting proteins from nuclear extracts. These samples can then be subjected to mass spectrometry to identify putative interacting proteins. (B) Protein ChIP-seq: Using antibodies targeting proteins of interest (or a tag), crosslinked chromatin from nuclei can be immunoprecipitated. DNA that is crosslinked at the site of protein binding can be de-crosslinked and sequenced, and then computationally aligned to a reference genome to reveal the genome-wide localization of the chromatin bound protein. These data can be compared to histone modification levels, as well as the enrichment of other proteins bound to chromatin. (C) Measuring histone PTM abundance and identification of novel PTMs. Following tissue dissociation, histones can be extracted using histone acid extraction protocols and then global levels of combinatorial histone PTMs can be assessed by mass spectrometry. This includes identification of potentially novel, brain-enriched histone PTMs. (D) CUT&RUN/CUT&TAG to map histone PTMs and chromatin binding proteins from low input. Nuclei isolated from dissociated brain tissue can be assessed for epigenetic modifiers/modifications from TFs. Novel methods include Cleavage Under Targets and Release Using Nuclease (CUT&RUN) or Tagmentation (CUT&TAG) sequencing, which employ pAG proteins to interact with antibodies that are bound to their targets in a native state, micrococcal nuclease (Mnase; CUT&RUN)) or transposase (TN5; CUT&TAG), followed by chromatin release. Subsequent sequencing allows for identification of genome-wide localization patterns of histone PTMs or nuclear proteins from low input samples. (E) Identification of DNA and RNA methylation sites and abundance. Utilizing specified sequencing methods, the locations DNA and RNA modifications, such as DNA 5-methylcytosine (5mC) or mRNA N6-methyladenine (m6A) can be identified. This allows for downstream determination of the effects of these modifications.

Faithfully implementing chromatin biochemical approaches to investigate epigenetic phenomena in brain is not a trivial undertaking, which perhaps explains why there are a limited number of studies characterizing detailed neuroepigenetic mechanisms in the context of neurological disease. This is partially due to the limited number of cell culture models for neural cells, the difficulty of performing genetic manipulations in primary neuronal culture systems, the cell-type (and circuit) heterogeneity of the intact brain, and the relatively low number of cells capable of being isolated from brains of model organisms for subsequent biochemical analyses (an “input” problem that is not typically encountered with genomic sequencing methodologies). These factors, among others, have greatly impaired the field’s ability to study the cell-type specific contributions of epigenetic modifications and chromatin/nucleic acid modifiers (along with TFs) using traditional biochemical methods. Methods used to interrogate epigenetic modifications and factors include immunoprecipitation of proteins coupled with mass spectrometry (IP-MS) for the identification of unique chromatin modifying complexes in brain and their components (Figure 3A), chromatin immunoprecipitation (ChIP), which maps genome-wide enrichment of histone modifications and TFs (Figure 3B), mass spectrometry for quantitative assessments of global levels of combinatorial histone PTMs (Figure 3C) (including identification of potentially novel, brain-enriched histone PTMs), as well as sequencing of DNA/RNA methylation levels (and sites).^124^ PRO-seq, ChIP-ReChIP, and 3D-genomic assays (Hi-C) are utilized to study transcriptional dynamics and chromatin architecture (detailed elsewhere;^125^^,^^126^^,^^127^), however, these techniques historically require millions of cells, and hence are limited by the low cellular yield from specific brain regions or even iPSC models. Such cellular “yield” problems using brain tissues places a massive constraint on researchers’ abilities to faithfully investigate biochemical roles for epigenetic modifiers in brain, as well as the consequences of their LoF or specific mutations.

### Modern assays allow for higher resolution epigenomics interrogations in brain

To deal with these cellular input problems, a number of novel methodologies have been pioneered over the past 5–10 years, which now allow for assessments of epigenetic modifiers/modifications and TFs from low input samples, such as brain. Two examples are cleavage under targets and release using nuclease (CUT&RUN) or tagmentation (CUT&TAG) sequencing, which employ pAG proteins to interact with antibodies that are bound to their targets in a native state. Activation of micrococcal nuclease (Mnase; CUT&RUN) or transposase (TN5; CUT&TAG), followed by chromatin release, allows for identification of genome-wide localization patterns of histone PTMs or nuclear proteins from low input samples (Figure 3B).^128^^,^^129^ Importantly, CUT&TAG was even recently pioneered for use at single-cell resolution for histone PTMs in brain and can effectively be combined with spatial sequencing technologies to assay the distribution of these modifications within specific brain regions.^40^^,^^130^^,^^131^

In addition to histone modifications and chromatin proteins, DNA and RNA modifications, particularly DNA 5-methylcytosine (5mC), DNA 5-hydroxymethylcytosine (5hmC) and mRNA N6-methyladenine (m6A), can also be examined at high resolution. For example, bisulfite sequencing has been used extensively to identify locations throughout the genome where DNA cytosines have been methylated (Figure 3E)^132^^,^^133^^,^^134^ Recent advancements have also developed bisulfite-free methods to identify DNA methylation at the single-cell level.^135^^,^^136^ Technologies for detecting mRNA m6A have been slower to develop, however, methods such as m6A-specific methylated RNA immunoprecipitation (MeRIP-seq) or m6A individual-nucleotide-resolution crosslinking and immunoprecipitation (miCLIP) have recently been reported (Figure 3E).^137^^,^^138^^,^^139^ These and other sequencing methodologies aimed at detecting changes in methylation have been reviewed extensively elsewhere, and are only beginning to be utilized in neurobiological research.^134^^,^^140^

To assess chromatin accessibility (or changes thereof) in brain, ATAC-seq is now the method of choice for most researchers. Utilizing a Tn5 transposase to cut and add sequencing adapters in areas of open chromatin, researchers can now explore at high resolution how chromatin accessibility changes in neural cells under different conditions.^36^^,^^141^ This strategy can be used to identify alterations in promoter accessibility as a result of treatment or protein mutations. Changes in accessibility can directly alter gene regulation through recruitment or exclusion of TFs. ATAC-seq can also be used to uncover cell-type specific (local vs. distal) enhancers that contribute to gene expression and are often aberrantly regulated in neurobiological disease states.^142^^,^^143^ Application of single-nuclei-based assays have been adapted for ATAC-seq, providing detailed, cell-type specific information regarding changes in local chromatin states. snATAC-seq can be coupled with snRNA-seq (commonly referred to as multiomic sequencing), allowing researchers to examine cellular heterogeneity in brain disorders, while simultaneously uncovering unique cell-types or cellular states (so-called “ensembles”) that result from human disease mutations. Recently, multiomic and CUT&TAG sequencing technologies have been further engineered to measure the spatial location of histone PTMs, chromatin accessibility and gene expression in a given cell/tissue, providing a more complete picture as to how transcriptomes respond to different genetic or environmental contexts^41^^,^^131^

### Mutations in ATP-dependent chromatin remodeler subunits are drivers of brain disorders

As mentioned earlier, there have been a multitude of mutations in ATP-dependent chromatin remodelers identified over the years. ATP-dependent chromatin remodelers play multifaceted roles in controlling histone occupancy, chromatin accessibility and ultimately transcriptional outputs in cells. Four main families of ATP-dependent chromatin remodelers have been described: SWI/SNF (BAF complex), INO80, ISWI, and CHD. These chromatin remodeler complexes are made up of multiple subunits, which increase the probability that mutations in one or more subunits may disrupt complex function (Figure 1G).^144^ SWI/SNF in particular is the most well characterized, with 3 known complexes identified: canonical BAF (cBAF), non-canonical BAF (ncBAF) and polybromo-associated BAF (pBAF). These distinct complexes contribute to both overlapping and distinct roles in chromatin regulation.^10^ For example, the cBAF complex has been found to harbor mutations in at least one subunit in over 20% of all human cancers.^145^ In addition, a recent study comprehensively characterizing cBAF complex mutations across many NDDs identified 2,539 cases who had a mutation in at least one cBAF subunit. In this study, they found that these mutations were predominantly heterozygous, due to the lethality of homozygous LoF variants. These variants consisted largely of point mutations in functional domains, protein-truncating mutations or other missense mutations leading to protein degradation.^10^

In certain NDDs, the core structural subunit of the BAF complex, *ARID1B*, was found to display the highest number of mutations among all complex members, with rodent studies corroborating the importance of ARID1B function in brain development. Mice with heterozygous LoF for *Arid1b* display intellectual disability (ID)- and ASD-like behaviors, along with deficits in inhibitory interneuron development.^146^^,^^147^ To understand how ARID1B disrupts brain developmental processes, extensive analyses, and mapping of mutations identified in humans with NDDs has been performed, with these mutations largely being found to be located within important functional domains, including within enzymatic and complex formation domains, as well as within intrinsically disordered regions (IDRs) of the protein.^10^ As demonstrated recently, IDR regions are important for BAF complex localization and function, and mutations in this region can consequently alter chromatin condensation and ATP-dependent chromatin remodeling activities.^148^ In addition, SMARCA2—the catalytic ATPase subunit of SWI/SNF—has been found to interact with the REST/NRSF complex and other schizophrenia risk genes. *Smarca2* knockdown in cortical neurons results in abnormalities in neurite length and dendritic spines, morphological phenotypes that are characteristic of many NDDs, including schizophrenia.^50^^,^^149^ Finally, as previously discussed, mutations in CHD proteins are also tied to a number of NDDs and other neurological disorders, and remain an active area of investigation.

In Down syndrome (Trisomy 21), the most common chromosomal condition leading to intellectual disability worldwide, the newly described BAF complex-associated factor BRWD1 (encoded on chromosome 21) was found to be significantly upregulated in patient-derived neurons and in brain tissues from a mouse model for Down syndrome (Ts65Dn)^150^^,^^151^ (Antonarakis et al. 2020; Fulton, Wenderski et al. 2022). Selective copy number restoration of *Brwd1* in trisomic mice was found to rescue transcriptional, synaptic and cognitive deficits in Ts65Dn mice,^151^ and biochemical investigations demonstrated that normal Brwd1 interactions with the BAF complex are disrupted in trisomic brain tissues leading to genome-wide mistargeting of the neuronal BAF complex and subsequent disruptions in chromatin regulation. These deficits were partially rescued by restoration of *Brwd1* copy number in trisomic animals, with over 65% of differentially accessible chromatin sites being restored. Such findings are of particular interest when considering future therapeutic treatments for Down syndrome, as restoring *BRWD1* copy numbers in trisomic human brain may prove useful as a therapeutic treatment.

### Chromatin modifying complexes have brain-specific variants that contribute to NDDs

One of the most well studied families of chromatin regulators are the polycomb repressive complexes (PRC1/2), which contain a number of variant proteins that differ based on auxiliary subunits to cf. specificity in targeting and activity in different cell-types and stages of differentiation.^152^ Some notable brain-specific examples are the non-canonical polycomb repressive complexes 1.3 and 1.5 (ncPRC1.3/1.5), which contain the brain specific protein AUTS2 and are linked to NDDs, such as ASD.^153^ Characterizations of these complexes have revealed that the casein kinase 2 protein inhibits ncPRC1 activity by phosphorylating RING1B at S168, adding an additional layer of regulation. AUTS2 was also found to bind to the HAT p300, leading to the activation of key neuronal genes that are important for normal mouse development, thus highlighting the complexity of these protein complexes in brain.^154^ Mutations in the AUTS2 HX repeat domain have also been found to result in neurodevelopmental deficits in humans, which overlap highly with those observed in Rubinstein-Taybi syndrome, a disorder caused by p300/CBP mutations.^74^

Additionally, there are multiple neuronal BAF complexes (as mentioned previously) with various subunits expressed in brain at specific time points, resulting in neural progenitor specific and mature neuron specific BAF (nBAF) configurations. For example, CREST (*SS18L1*), a calcium-regulated transcriptional activator within the nBAF complex, has been found to be mutated in ALS,^52^ with these mutations disrupting its interaction with the HAT CBP.^51^ Transgenic mice with these *CREST* mutations exhibit CNS protein instability, heightened inflammatory responses, microglia activation and motor deficits, consistent with the human condition.^155^ ChIP- and RNA-sequencing experiments further revealed that the nBAF complex represses chemokine genes through HDAC1 recruitment to chromatin in neurons,^155^ and as such, mutations in *CREST* have been hypothesized to disrupt this repression, leading to immune activation and motor deficits.^156^ Assessments of these brain specific chromatin modifying and remodeling complexes, which are often mutated in brain disorders, have highlighted differences and heterogeneity in brain in comparison to other tissues, uniquely functioning to modify transcription.

**Figure 4:**
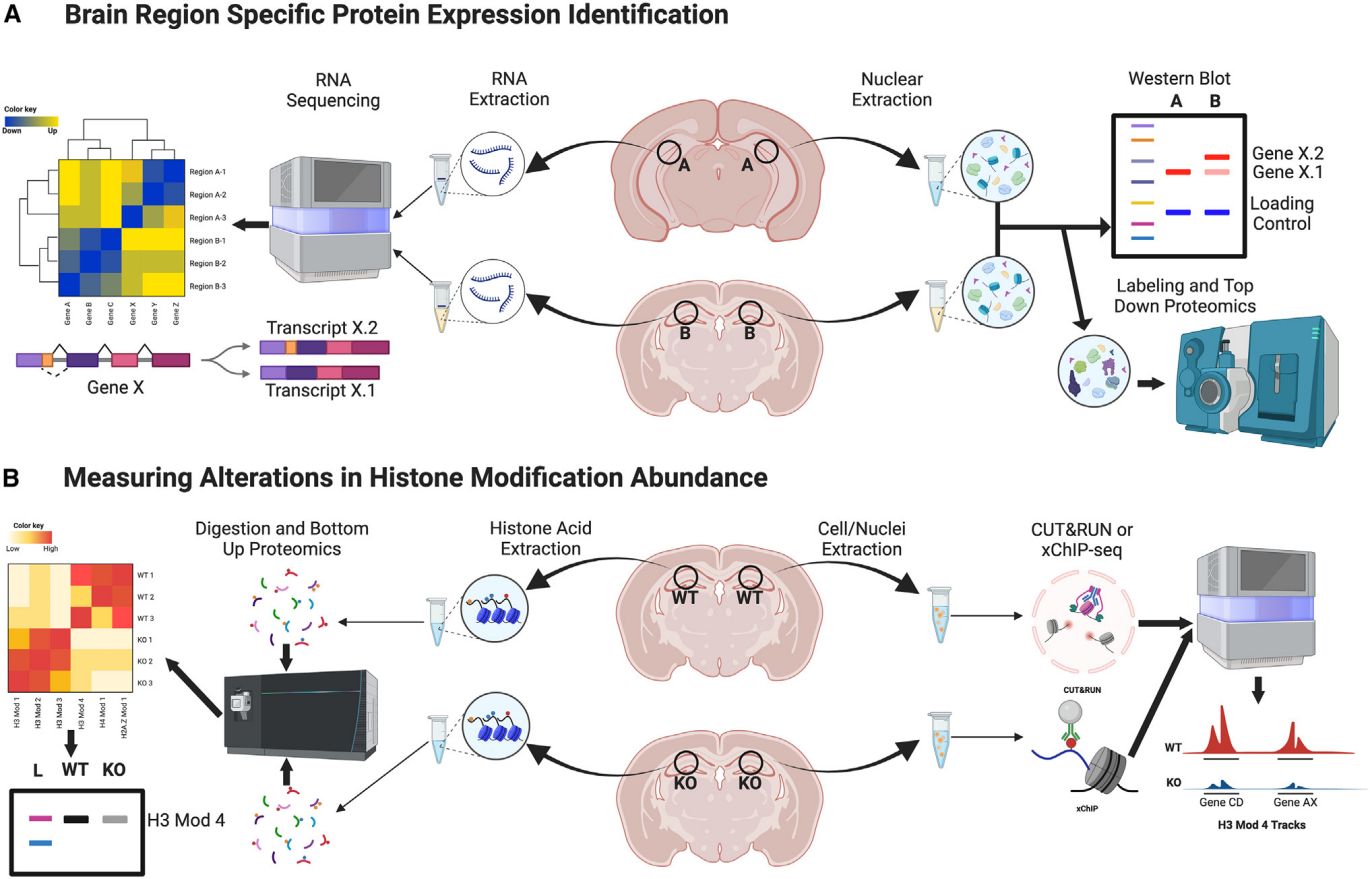
Methods to characterize epigenetic modifiers and histone modifications in brain (A) Brain region specific protein expression identification: Tissues from specific brain regions can be collected from rodent brains or dissected from human postmortem brains. RNA or protein extracts can be prepared from tissues and used for downstream analyses. Following RNA sequencing, expression levels of epigenetic modifying enzymes and “readers” can be quantitatively compared across brain regions. Additionally, alternatively spliced transcript expression can be assessed from RNA-seq data. Protein levels and varying isoform expression in different brain regions can be assessed by western blotting or mass spectrometry. (B) Measuring alterations in histone modification abundance: Tissues from specific brain regions can be dissected from WT or transgenic rodents, or could be compared between a WT vs. diseased patient postmortem tissues. Histones can be acid extracted, digested into small peptides, and loaded onto a mass spectrometry instrument. Following MS, quantitative analysis of the levels of multiple histone PTMs can be assessed in combination. Differences between conditions can be confirmed by western blotting of histone PTMs from the same tissues. Cells and/or nuclei can additionally be isolated and used for either crosslinked (x) ChIP-seq or CUT&RUN(or TAG) using antibodies that are specific for the histone modification under study. Using spike-in controls, levels of these modifications at all loci can be quantitatively compared between samples.

Through increasingly sensitive mass spectrometry and RNA-seq methods, brain-region and time-specific characterization of RNA and protein levels can be quantitatively measured (Figure 4A). This approach is vital to understanding the intricacies underlying the heterogeneity and uniqueness of brain-specific variants and time/region sensitive expression. While much still remains unknown regarding the precise mechanistic actions of individual ATP chromatin remodeler complex members or their roles in brain disorders, gaining a clearer mechanistic understanding of these complexes in brain promises to reveal intricate mechanisms contributing to neurodevelopment, which may additionally allow for targeted therapeutic strategies aimed at restoring normal ATP-dependent chromatin remodeling complex activities in NDDs and other diseases.

### Histone variants are uniquely expressed in brain and are vulnerable to mutations

Neurons in brain represent a unique cell-type within the human body, with humans developing nearly all of their neurons by 18 months of age.^157^ Other tissues continue to divide and grow into adulthood, and cells continuously undergo replication-dependent histone turnover. The lack of cell replication during neurodevelopment suggests that histone deposition and eviction in brain must be largely regulated independently of replication, making neurons unique in comparison to actively proliferating cells in other tissues. This occurs via chaperone-mediated histone turnover, whereby replication-independent histone variants are incorporated into chromatin to replace the canonical histones reviewed here in the study by Gurard-Levin et al.^158^ It has been hypothesized—due to evidence obtained from proliferating cell populations—that histone variants (i.e., H3.3, H2A.Z, MacroH2A, etc) serve as epigenetic “barcodes,” effectively bookmarking domains of chromatin for specific functions.^159^^,^^160^ In post-mitotic cells of the brain, on the other hand, it was widely believed that histone turnover events are largely static, leading to the existence of long-lived histone proteins in neuronal chromatin.^161^^,^^162^ Recently, however, it was demonstrated that the histone H3 variant H3.3, which is deposited in a replication-independent manner, essentially replaces all canonical H3 histones (H3.1 and H3.2) during neurodevelopment, reaching near-saturating levels throughout the genome by mid-adolescence in humans and rodents (Figure 1H).^107^ This phenomenon then allows for the continuous, and highly dynamic, turnover of histone H3.3 (and thus presumably nucleosomes as a whole) in neurons and glial cells by the H3.3 chaperone HIRA throughout life, which was shown to play important roles in synaptic connectivity during development. This turnover, while highest during neurodevelopment, continues into adulthood and can be induced by neural activity to promote neuronal plasticity, thus contributing to many long-term physiological and behavioral processes, including learning and memory.^107^

Later, it was demonstrated that aberrant H3.3 dynamics in nucleus accumbens (NAc), a key limbic reward region of the brain, contributes to the regulation of mood dynamics and stress-induced behaviors in mice. Combatting such aberrant dynamics by suppressing the expression of both H3.3 genes (*H3f3a/b*), which stalls histone turnover in neurons, rescued stress-induced gene expression and behavior.^163^ H3.3 was also shown to accumulate at key genes in response to cocaine self-administration in mice in concordance with their increased transcription, further highlighting the dynamics of histone turnover in response to external stimuli.^164^ As previously discussed, H3.3 is encoded by only two genes (*H3F3A/H3F3B*) in the human genome, making it vulnerable to consequential mutations. Indeed, there have now been extensive studies of germline H3.3K27M mutations and their roles in both diffuse midline gliomas and diffuse intrinsic pontine gliomas in brain, with these mutations resulting, at least in part, in inhibition of PRC2-mediated methylation. This, in turn, leads to a global redistribution of H3K27me3 (a repressive histone PTM), inappropriate patterns of gene regulation and the emergence of cancer-related phenotypes.^165^^,^^166^^,^^167^ Such “oncohistones” have become the focus of extensive study in cancer neuroscience and represent viable targets for the treatment of these deadly pediatric cancers.^168^^,^^169^ In addition, the aforementioned H3.3 germline mutations in NDDs likely contribute to transcriptional disruptions via distinct mechanisms. For example, one of the mutations, H3.3G34R, was recently found to disrupt the recruitment of the DNA methyltransferase, DNMT3A, thereby reducing genomic DNA methylation levels and inhibiting H3.3K36me2 on mutated histones.^170^

Other histone variants have also been shown to contribute uniquely to gene expression in brain vs. non-neural tissues. For example, the histone H2A variant H2A.Z was demonstrated to be required for proper neurodevelopment and consolidation of memories within the hippocampus.^171^^,^^172^^,^^173^^,^^174^ In addition, the H2B variant H2BE, was found to be regulated in olfactory neurons in an activity-dependent manner,^175^ suggesting a role in olfactory processing. Comprehensive characterizations of H2BE have demonstrated ubiquitous expression in neurons throughout the brain, with enrichment at gene promoters and high correlations with chromatin accessibility. Indeed, H2BE was shown to be required for the proper accessibility and expression of key synaptic genes during development, with genetic KO of H2BE significantly disrupting long-term memories in mice.^176^ Such unique roles for histone variants in brain indicate the necessity of comprehensively studying epigenetic modes of regulation within neural cells specifically (vs. proliferative cells, for example), as these mechanisms of action are often divergent from other tissues. Through utilization of RNA-seq and mass spectrometry methods, researchers can unbiasedly assess various brain regions at different time points (i.e., during development or during aging) for changes in the expression and presence of histone variants (Figure 4A). Thus, extensive characterization of histone variants and their mutations in both neurons and non-neuronal cells in brain is needed, as the resulting impact of these mutations in post-mitotic cells may differ from that of other somatic populations.

### Novel histone PTMs impact transcription in brain in multiple contexts

Since the beginning of the epigenetics boom and the chromatin field’s proposal of a “histone code hypothesis” by David Allis and colleagues over 20 years ago, the field of epigenetics has attempted to identify all histone PTMs and their roles in transcriptional activation or silencing.^177^ To date, there have been dozens of novel modifications discovered in addition to the most commonly found and interrogated histone PTMs, such as acetylation, methylation, and phosphorylation.^5^^,^^6^ In the context of brain cells, these well characterized modifications have been studied, but to a lesser degree from a mechanistic standpoint. It is now evident that many of the most commonly described histone modifications, such as acetylation, methylation, etc., play critical roles in guiding transcriptional plasticity in the CNS. Through groundbreaking studies in recent years, however, it has also become clear that there exists a larger plethora of potentially novel histone PTMs (using alternative metabolic donors), which may be enriched in brain and can play critical roles in transcriptional regulation and processes associated with neurodevelopment and brain disorders. These PTMs include, but are not limited to, histone butyrylation, lactylation, ADP-ribosylation, and crotonylation, the latter which has been shown to be reduced in response to chronic social stress in the prelimbic cortex, a brain region associated with depressive-like behaviors (Figure 1A).^5^^,^^178^ Another such class of modification is that of histone monoaminylations, which occur at histone H3 glutamine (Q) 5, whereby linear hydrophobic monoamine neurotransmitters, such as serotonin, dopamine, and histamine, can serve as chemical donors for the covalent modification of histones by the tissue transglutaminase 2 (TGM2) enzyme.^179^^,^^180^^,^^181^^,^^182^^,^^183^ These modifications can be permissive (serotonylation and dopaminylation) or repressive (histaminylation) in the regulation of neuronal transcription, mediated, in part, via altered interactions with components of transcription factor complexes, such as TFIID, as well as histone methyltransferase complexes (e.g., MLL1) and demethylases (e.g., KDM5 and LSD1).^179^^,^^181^^,^^182^ These monoaminylation modifications exist at varying levels across subregions of the brain and have been shown to play critical roles in neural differentiation/development,^179^^,^^184^ sensory processing,^185^ cocaine and heroin dependence,^180^^,^^186^^,^^187^ stress and depression-related phenotypes,^188^ and sleep/wake cycle regulation.^183^ As these new modifications are investigated more extensively, and perhaps even more are discovered, it will be important to identify the entire repertoire of their potential *readers*, *writers*, and *erasers* in brain (as well as in peripheral systems), as it might be predicted that mutations in monoaminylation associated proteins will cf. susceptibility to neurological and/or psychiatric disease. Any identified *readers* may also serve as novel targets for the treatment of related disorders, and as such, gaining a more complete understanding of these complex mechanisms holds promise for future therapeutic development.

### Histone PTMs in brain are altered in various physiological states through complex mechanisms

As articulated in the “histone code hypothesis,” histone PTMs can influence gene expression through interactions with chromatin *readers*. While not a binary code, the interplay of *reader* domains and histone PTMs are thought to drive transcriptional processes (i.e., activation, silencing, and splicing) via both *in cis* and *in trans* mechanisms. In brain, complex and dynamic interactions between *readers* and histone modifications are presumed to occur in a cell-type and regional-specific manner, with these interactions likely influenced by distinct environmental contexts. Disruptions in these mechanisms are not always due to mutations in chromatin-related proteins, but can be due to other genetic changes (i.e., in signaling proteins and/or in non-coding regions) or to external stimuli (e.g., exposures to stress or drugs of abuse). A number of recent studies have begun to thoroughly characterize changes in histone PTMs and their enzymatic machinery in the regulation of neurological disease states and in response to external stimuli. For example, using a multiomic approach, Nativio et al. identified widespread changes in histone modifications in AD using tissues from postmortem human brains through utilizing histone mass spectrometry from brain tissue (Figure 4B). In this study, they identified increases in H3K9ac and H3K27ac in AD, which were concomitant with the histone acetyltransferase CBP/p300 being significantly upregulated in affected individuals. They were further able to link these alterations in histone modifications to disease-specific genes using ChIP-seq, thereby elucidating the specific transcriptional pathways that are controlled by these PTMs, which appear to contribute importantly to the progression of AD.^189^ Another recent study by Farrelly et al. examined histone modifications in a hiPSC derived neuronal model of schizophrenia. Using bottom-up mass spectrometry to compare combinatorial histone PTM profiles in schizophrenia vs. healthy control neurons, significant increases in combinatorial acetylation of both the histone H2A variant H2A.Z, and histone H4 were identified. Such elevations in H2A.Zac in SZ neurons were found to result in the increased binding of BRD4, which was shown to be a *bona fide reader* of triply acetylated H2A.Z, thereby leading to increased expression of schizophrenia-related genes. Importantly, it was found that this interaction was targetable for the restoration of normal gene expression using the broad BET inhibitor JQ1.^190^ Studies such as these have begun to illuminate mechanistic interactions between *writers*/*readers* of histone PTMs, which, when imbalanced due to disease states, can lead to long-lasting effects on transcription—and therefore cellular function—leading to disease progression.

There are, of course, limitations to using postmortem brain tissues and hiPSC derived neural cell models, as in the above studies, which often include limited sample sizes, a lack of etiological control for *in vivo* environmental impacts and genetic background, among others. Rodent models, while never fully being able to recapitulate human disorders, allow for better control over these factors. For example, using rodent models, Kronman et al. investigated histone modifications (using methods like in Figure 4B) in the NAc following chronic exposures to early life stress (ELS), where they observed reduced levels of H3K79me1/me2 in adult male mice. This was concurrent with increased expression of both the H3K79me *writer* (Dot1l) and *eraser* (Kdm2b) enzymes in dopamine D2, but not dopamine D1, receptor-expressing medium spiny neurons (MSNs). Further ChIP-seq analyses revealed that while H3K79me2 is globally decreased genome-wide following ELS, locus-specific increases in the mark contribute importantly to stress-induced gene expression. Inhibiting *Dot1l* reversed ELS-induced behavioral deficits and rescued stress-mediated gene expression.^191^ In a related study, Torres-Berrío et al. used the same model of ELS to identify elevated levels of H3.3K27me1 in NAc D1 MSNs following chronic social defeat stress in mice. There, they found that SUZ12 (the *writer* for H3.3K27me1) increased expression after chronic stress, resulting in increased enrichment of H3.3K27me1 within genes linked to aberrant stress-induced neuronal excitability. Overexpressing SUZ12’s C-terminal VEFS domain in D1 MSNs specifically increased H3.3K27me1, leading to heightened stress susceptibility and intrinsic neuronal excitability.^192^ These studies nicely highlighted potential links between environmental risk factors and lasting epigenetic changes, findings that may serve to complement or inform studies focused on human disease. They also underscore the importance of regional and/or cell-type-specific histone PTMs assessments in models of human disease, findings that may pave the way for more targeted therapeutics in the future. Finally, understanding the sequence of developmental gene activation pathways unique to the brain will be critical for developing effective therapeutic strategies during these dynamic neurodevelopmental windows.

### Nucleic acid modification disruptions affect transcription and translation in brain disorders

DNA and mRNA modifications, in particular methylation and hydroxymethylation, also represent an emerging area of interest in the field of neuroepigenetics. While there are a plethora of known DNA and RNA modifications, only a select few (DNA 5mC/5hmC, and mRNA m6A) have been well characterized. These modifications can directly affect other processes, such as TF binding to DNA, RNA stability and localization, and RNA alternative splicing.^193^^,^^194^ DNA methylation itself has long been known to be disrupted in neurological diseases, particularly in the context of aging, but there have also been well-characterized examples of mutations in DNA modifying enzymes and DNA methylation binding proteins.^8^^,^^195^ Mutations in MECP2, the methyl-binding protein MBD5 and the DNA methyltransferase DNMT3A all contribute to NDDs. Select studies have further explored the mechanisms through which DNMT3A haploinsufficiency and MBD5 LoF affect neurodevelopment,^196^^,^^197^^,^^198^^,^^199^ and knockout and patient-specific mutation knock-ins of *Mecp2* in rodent models have recapitulated patient relevant behavioral and motor impairments, thus allowing for future studies aimed at uncovering the molecular mechanisms driving these phenotypes.^200^^,^^201^^,^^202^

Similarly, the mRNA m6A modification has recently received much attention in the field, with novel brain specific roles for this PTM now having been uncovered. For example, it was found that m6A abundance increases throughout brain development and is required for proficient neurogenesis, as well as being required for the proper subcellular localization of select neuronal mRNAs in hippocampal neurons.^137^^,^^203^^,^^204^ Additionally, m6A methylation of mRNA was found to be destabilized in ALS iPSC models and in postmortem spinal cords from C9ORF72-ALS/FTD and sporadic ALS patients, respectively, demonstrating a global decrease in the former and a global increase in the latter. These studies highlight how resulting changes in the m6A modification affects RNA stability through interactions with RNA-binding proteins and their downstream pathways, which can lead to decreased neuronal survival.^205^^,^^206^ Correcting imbalances in the levels of RNA methylation significantly improves the survival of neurons in both models. Collectively, these studies demonstrate that disruption of DNA methylation, DNA-methyl interacting proteins, RNA modifications, and RNA-binding proteins all can affect neurodevelopment and neuroplasticity, similar to that of histone PTM and chromatin remodeling pathways.

## Bridging the gap between genomic and biochemical understandings of neuroepigenetic phenomena

### Epigenetics studies of cancer and stem cells: Paving the way for therapeutic approaches for brain disorders

Mechanistic biochemical research on histone PTMs, their *readers*/*writers*/*erasers*, chromatin remodeling enzymes, and DNA/RNA methylation in other fields, such as cancer biology and stem cell biology, have led to detailed understandings of the mechanisms underlying a plethora of diseases, particularly with respect to a wide range of human cancers. Identification of tumor suppressor or oncogenic functions of these epigenetic modifications and modifiers has led to many successful attempts to use drugs to target these mechanisms in cancer patients. To date, there are number of FDA approved drugs targeting epigenetic modifiers, aimed at inhibiting DNMTs, HDACs, and the PRC2 subunit EZH2 for various cancers. There are hundreds of more drugs (and alternative strategies, see section “epigenetic drugs and delivery methods need to be developed for targeting brain disorders”) undergoing development and testing, as well as clinical trials testing combinatorial therapies.^207^^,^^208^ These successes are due to decades of research on these epigenetic complexes, and mechanistic studies into their roles in cancer progression. With an ever-expanding toolkit now available, the field of neuroepigenetics is hoping to follow the cancer field’s lead toward understanding the underlying epigenetic mechanisms of a multitude of brain disorders, which promises to one day yield viable therapeutic options for patients.

### Employing chemical biology tools for the study of histone modifications in brain

**Figure 5:**
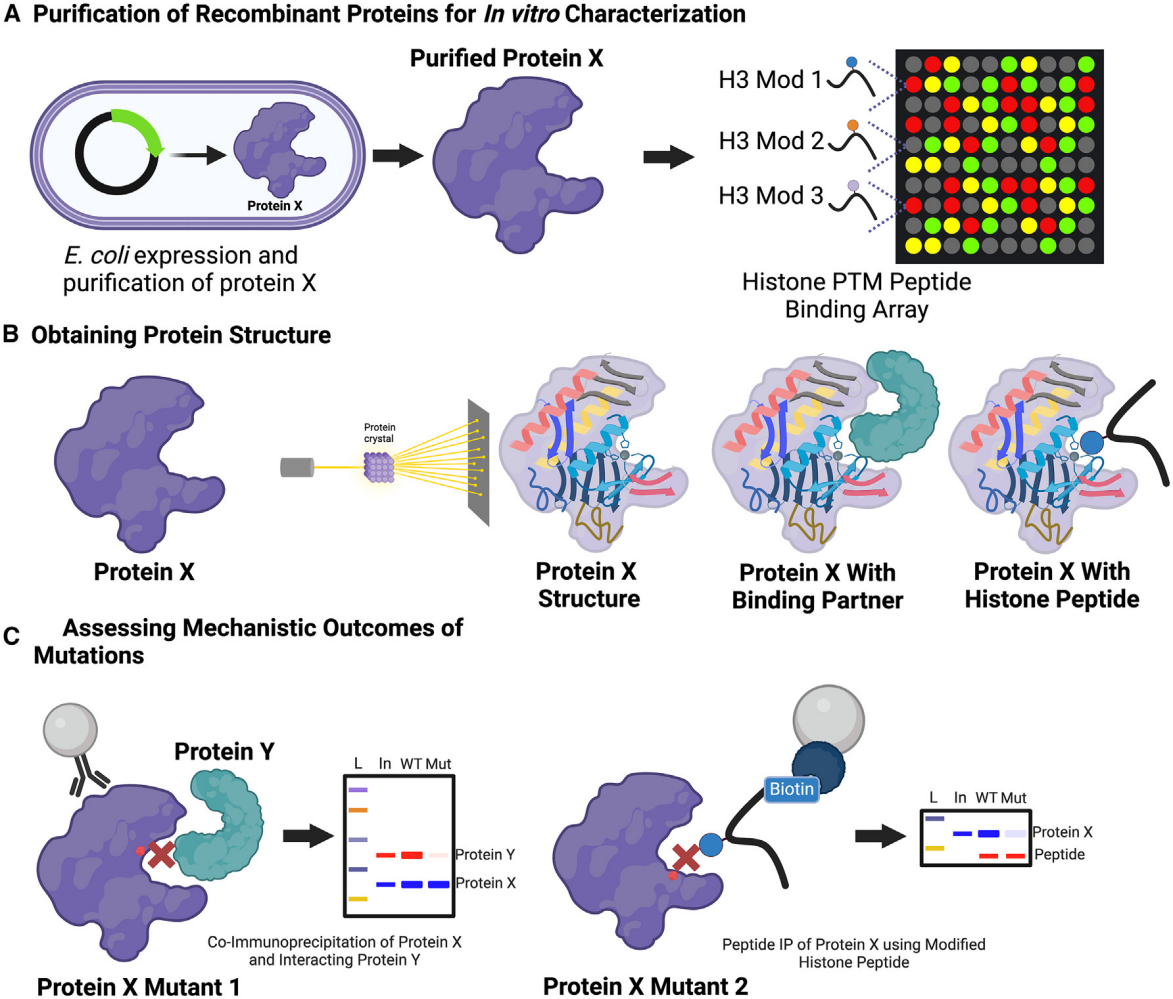
*In vitro* methods to assess chromatin regulatory protein structure and function (A) Histone modification peptide binding arrays: A plasmid containing a protein of interest’s cDNA and a tag (such as 6X-histamine) can be transformed into *E. coli*, and proteins can be expressed and purified using affinity strategies against the tag (Nickle affinity beads for 6X-histamine, for example). Purified proteins can then be loaded onto histone PTM peptide binding arrays, where each well contains a peptide with a distinct PTM or combination of PTMs. Quantitative blotting is then carried out for the tagged protein to reveal which modifications the protein preferentially binds. (B) Obtaining protein structure: The same recombinant proteins from C can be used to seed growth of protein crystals, which then can undergo X-ray crystallography, with the results of these experiments used computationally to build high-resolution 3D structures. Additionally, the structure of the protein with known binding partners and histone peptides with select modifications can be obtained. (C) Assessing mechanistic outcomes of patient associated mutations: Following acquisition of the structure of a given protein of interest from B (which can inform on key amino acid residues important for binding and/or activity), or using clinical mutations, mutant proteins can be expressed *in vivo* or in *E. coli* and used for downstream characterizations. The impact of these mutations on binding partners can be measured by co-IP experiments, and the impact of these mutations on binding to histone PTMs can be measured by comparing peptide IPs of wild type vs. mutant proteins or other precise methods to measure binding affinity.

To mechanistically study the causal impact of histone PTMs in brain, the development and implementation of novel genetic and chemical tools/approaches will be needed to directly manipulate histone modifications *ex vivo* and *in vivo* with limited off-target effects. One manner through which this has been achieved is through viral vector-mediated expression of wild type vs. mutant H3.3 in brain, the latter incorporating point mutations that effectively ablate endogenous modifications in a dominant negative fashion (Figure 5A).^163^^,^^179^^,^^209^ This approach, while effective, is limited, however, since the rates of chromatin incorporation of exogenously expressed mutant H3.3 proteins are often low in comparison to endogenous H3.3 levels, and the expression of mutant histones can also cause off-target effects by impacting cross-talk with other histone PTMs and subsequent downstream transcriptional consequences. It is thus critical to control for exogenous histone incorporation by including both WT and mutant variants in experimental designs. Of note, such mutational approaches could also be implemented in human IPSC neuronal model systems (e.g., mutating both *H3F3A/H3F3B* simultaneously without the need for overexpression), as differentiation of mutated IPSCs into neurons would then result in a pure population of cells with the desired mutation. In addition, epigenetic modifiers, such as G9a/EHMT2 or p300, and their related downstream effects, can be targeted using locus-specific approaches, such as those that use engineered TFs (e.g., zinc-finger proteins/ZFPs)^210^ or transcription activator-like effectors (TALEs).^211^ The use of catalytically dead Cas9 (dCas9) fusion proteins is an even more recent tool that has been developed for site-/locus-specific manipulations of histone modifications in brain.^212^ This technology was successfully applied to investigate causal roles for H3K36me3 in aberrant alternative splicing following chronic cocaine exposures.^213^ By fusing the H3K36 methyltransferase Set2 to dCas9, researchers were able to target this fusion protein to a specific alternatively spliced locus in NAc, resulting in increased H3K36me3 deposition.^213^ Such mechanistic experiments are important in demonstrating causality for epigenetic modifications at specific loci in development and disease. An important limitation to note, however, is that the fusion of catalytic domains to a tethering protein may not be representative of biological conditions and could therefore lead to a loss of amino acid substrate specificity of the *writers* under study. Rigorous validations to ensure a lack of off-targeted effects must be performed in order to appropriately draw conclusions, and additional *in vivo* experiments should be performed to confirm results.

Intein chemistry exists as another rapidly evolving chemical methodology that offers promise in delivering site-specific histone PTMs in cells for downstream causal assessments. For example, a truncated form of histone H3.3 fused with a C-intein on its N-terminus can be co-expressed in cells or tissues (including brain) with engineered peptides consisting of the H3 tail (containing PTMs of choice) with an N-intein fused to the C-terminus. The respective C- and N-inteins can then be spliced together in a traceless manner to *de novo* generate site-specific histone PTMs *in vivo*. This approach has been used to assess the contributions of specific histone PTMs to the establishment of other histone modifications, interactions of *reader* proteins and transcriptional outputs.^214^^,^^215^ Generation of these “designer” nucleosomes carrying site-specific specific PTMs allows for cross-talk interaction studies between histone PTMs and their associated *reader* proteins.^216^^,^^217^ To date, this approach has primarily been used in cell lines and was only just recently tested for use in live-animals using brain-region specific viral delivery of truncated H3.3-intein fusion proteins in mouse NAc, followed by intra-brain delivery of intein-fused peptides; these experiments provided a potentially exciting new technology for further characterization of histone PTMs in brain.^218^

### The importance of identifying epigenetic protein structures, modifications, and physical interacting partners

Despite extensive characterizations of identified risk genes using rodent and human cellular models, many questions persist regarding specific cellular and/or molecular outputs of disease-causing protein mutations, and the resulting impact of these mutations on altered transcriptional states in disease. Specifically, the impact of these phenomena on protein-protein interactions and/or structural changes that may be induced to precipitate disease remains unclear. *in vitro* assays using purified protein (WT vs. mutant) from *E. coli* can be used to measure the functional consequences of these mutations. Peptide PTM binding arrays can be used to screen WT vs. mutant proteins to see which histone PTMs they preferentially bind to (Figure 5A). More modern methodologies, such as cryo-electron microscopy (cryo-EM) or X-ray crystallography, can map protein structures at atomic resolution, and the recently developed Alpha-Fold software allows for computational predictions of complex protein structures and interactions with high accuracy (Figure 5B). These sites can then be used to predict and test (*in vitro*) impact of disease-related mutations on protein function and binding to histone PTMs (Figure 5C).^219^^,^^220^ Coupling this with known binding partners of the protein of interest (Figure 4), these *in vitro* methods can be applied to test how these point-mutations may disrupt interactions with their binding partners (Figure 5C). With all of this information obtained, human iPSCs lines and transgenic mouse models can be generated with either disease-relevant, lack of binding, or lack of activity mutations to test the impact of disruption of binding kinetics of the protein of interest on gene expression profiles, the chromatin landscape, and ultimately behavioral phenotypes (Figure 4). Mass spectrometry can also be utilized to quantitatively measure histone PTM levels in order to determine brain region specific changes in histone modifications during development and in disease (Figure 3B). Bottom-up proteomics captures data from short peptide sequences in order to offer high-resolution insights into PTMs and can be implemented to study PTMs in brain and other cell-types (Figure 3B). Top-down proteomics, on the other hand, investigates entire histones without protein digestion, enabling identification of protein isoforms, histone variants and analysis of all PTMs on a given histone (so-called proteoforms) from specific brain regions (Figures 3A and 3B), but at lower resolution. Despite its lower resolution, top-down proteomics can provide a more complete understanding of combinatorial PTMs, which may prove beneficial to studies of histone PTM regulation in brain, particularly in the context of disease. While technically challenging, middle-down proteomics serves as a compromise, displaying high accuracy and success in recent years; all of these techniques are reviewed extensively elsewhere.^221^

Once PTMs (and their regulation by environmental stimuli or in disease states) are identified, customized or designer recombinant nucleosome arrays can then be used to gain further mechanistic insights *in vitro*. For example, synthesized H3K4me3 nucleosomes were used to show that KDM5A (a lysine demethylase) activity is stimulated by the binding of the plant-homeobox domain 1 (PHD1) finger to unmodified H3 tail peptides, inducing a conformational change that allows the jumonji C (JmjC) domain to de-methylate H3K4me3.^222^ Chemically assembled designer asymmetric nucleosomes (containing one H3unmod and one H3K4me3) confirmed that KDM5A functions to resolve asymmetric methylation on a nucleosome, as the absence of methylation on one histone octamer increases the activity of KDM5A on the other, as well as (albeit to a lesser extent) on neighboring nucleosomes.^223^ Designer nucleosomes can additionally be utilized to discover novel *readers* for specific histone PTMs, including for novel histone modifications identified in brain, such as monoaminylations.^224^^,^^225^ All of these methods can then be integrated with the previously discussed molecular technologies, such as inteins or dCas9 targeting, to better understand the biochemical mechanisms underlying brain (dys)regulation. These methods are rapidly evolving due to the continued efforts of chemical biologists and epigenetics, and have recently been reviewed here in the study Hegazi et al.^226^

## Exciting new technologies and therapeutic approaches to target neuroepigenetic dysregulation in brain disorders

### Epigenetic drugs and delivery methods need to be developed for targeting brain disorders

Epigenetics-based therapies have largely been deployed thus far to treat cancer, and they primarily target histone modification *readers*, *writers*, and *erasers*.^227^ These drugs are often broad-acting and influence the activity and/or binding of multiple proteins, which is not necessarily ideal for targeted treatments. Furthermore, to treat brain disorders, drugs need to be able to cross the blood-brain barrier (BBB) and reach a given brain region of interest, which presents its own set of complications. Select FDA-approved drugs for treating epilepsy, schizophrenia, and bipolar disorder include valproate products, some of which act as HDAC inhibitors in addition to their primary pharmacological actions.^228^^,^^229^^,^^230^ In the case of major depressive disorder (MDD), selective serotonin reuptake inhibitors (SSRIs)—which increase serotonin tone by blocking its reuptake by the serotonin transporter, SERT—represent one of the most commonly prescribed classes of drugs on the market for the treatment of affective disorder.^231^ However, recent findings indicate that histone H3 serotonylation can be reduced by SSRIs, particularly in serotonergic regions of the brain where H3Q5ser is dysregulated in patients with MDD (e.g., dorsal raphe nucleus [DRN]). These drugs may function, at least in part, through alterations of this newly discovered PTM. By identifying these potentially indirect pharmacological actions of SSRIs in people with MDD and other mood-related disorders, the field may be able to gain a better understanding of how these drugs affect epigenetic processes to alleviate depressive symptoms. Treatments with chronic fluoxetine (an SSRI), for example, were found to significantly reduce the aberrant stress-induced accumulation of H3Q5ser in DRN of mice.^188^ Furthermore, it was determined that attenuating stress-induced accumulation of H3Q5ser using mutant H3.3Q5A histones was sufficient to promote resilience to chronic social stress and rescued stress-mediated gene expression. This work highlights the urgent need to better understand how current psychiatric treatments impact the epigenome, ultimately leading to long-term changes in transcriptional plasticity, circuit function and behavior. This can occur directly via inhibition of epigenetic modifiers, or indirectly by altering the gene expression of epigenetic *writers*/*readers*/*erasers* and/or the local concentrations of monoamines (or other donor metabolites; e.g., acetyl-CoA and SAM) in neural cells.^232^^,^^233^^,^^234^^,^^235^ Since antidepressants can take weeks to relieve symptoms, this knowledge could guide treatment strategies and enable more efficient interventions targeting the underlying epigenetic processes of these disorders. In future drug development efforts, it could be that supplementing “standard of care” drugs for brain disorders with epigenetic activators/inhibitors may help to boost patient responsiveness, as such strategies have proven successful as a therapeutic avenue for many cancer subtypes.^227^^,^^236^

**Figure 6:**
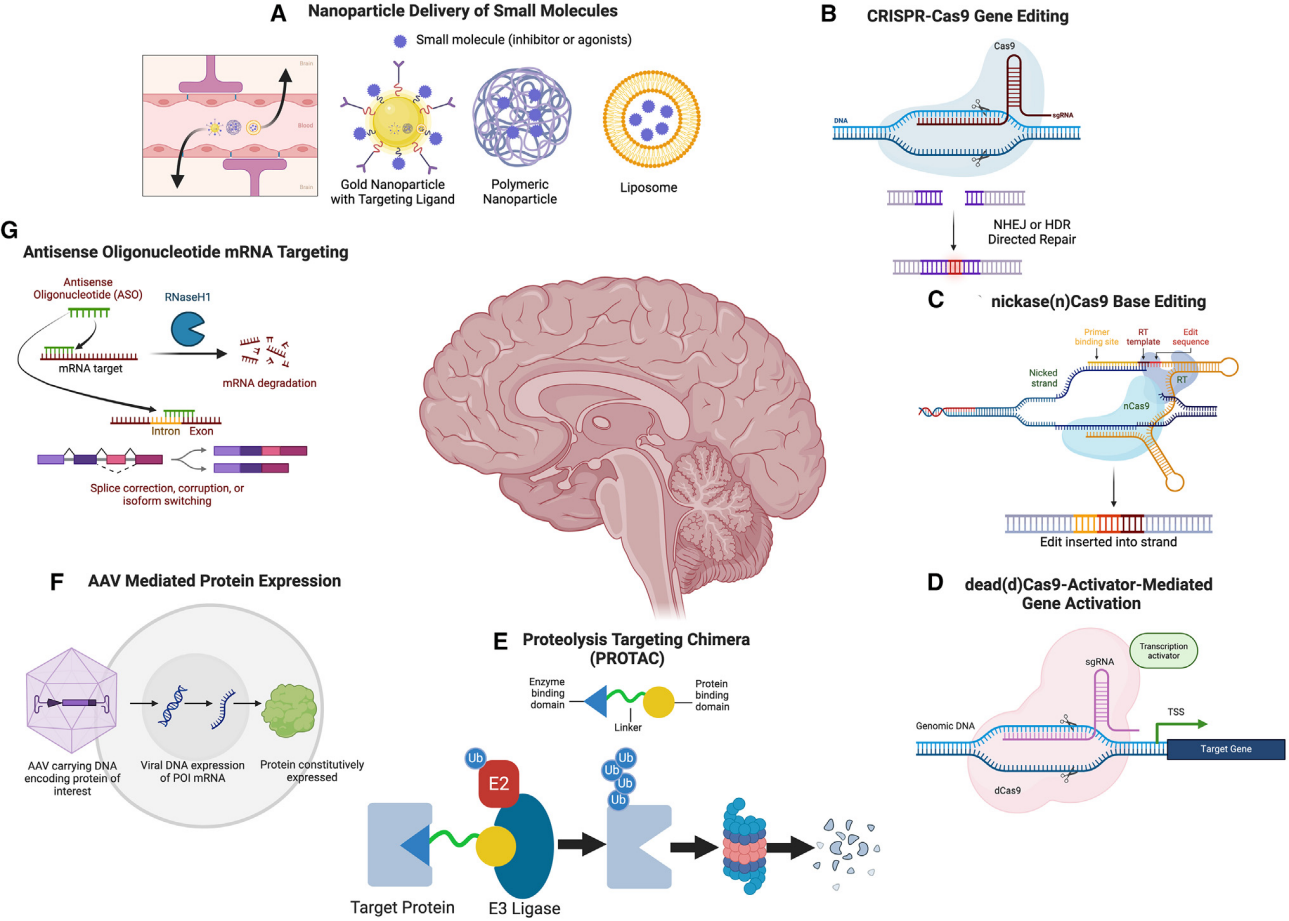
Methods for targeted epigenetic therapies in brain (A) Nanoparticle delivery of small molecules: a comprehensive toolbox of nanoparticles have been developed, which can be utilized to deliver small molecule inhibitors or protein antagonists to the brain. They have been shown to robustly cross the blood-brain barrier. (B) CRISPR-Cas9 gene editing: CRISPR-Cas9 can be delivered to the brain for editing of the genome at a desired locus. This approach can be used to correct patient associated mutations, alter splicing patterns, or knockout of a given gene of interest. (C) Nickase Cas9 base editing: the Cas9 nickase enzyme can be used to selectively nick one strand of DNA for base or prime editing. This approach allows for precise (but minor) edits of DNA and avoids relying on DNA double stranded breaks (DSBs) or homology-directed repair, which results in lower off-targeted effects. (D) Catalytically dead Cas9 activator mediated gene activation: dCas9 can be fused to a transcriptional activator, such as VPR, and targeted to a region of interest near a specific gene’s TSS. This approach can result in the activation of a gene that is lowly expressed (or not at all). (E) Proteolysis targeting chimera: a small molecule or peptide targeting a protein of interest can be linked to an E3 Ligase protein binding domain. When bound to the target protein, it then recruits an E3/E2 Ligase, which polyubiquinates the protein leading to proteasomal degradation. This approach can be used to target proteins that are overexpressed, have hyperactive activity, or are impeding other proteins’ activity in brain disorders. (F) Adeno-associated virus (AAV) mediated protein expression: AAV vectors with DNA encoding a protein of interest can be delivered into brain. Certain AAVs can be used to target the brain specifically, and tissue-specific gene promoters can be used to improve specificity. This approach can be used to rescue the protein expression of proteins that may be rendered inactive or not expressed in a given brain disorder. (G) Antisense oligonucleotide (ASOs) mRNA targeting: ASOs can be designed to target the mRNAs for a protein of interest. This approach can be designed to lower the protein’s expression levels or introduce splicing changes in order to achieve an alternatively spliced transcript. ASOs can also be delivered easily across the blood-brain barrier.

Increasing mechanistic insights into roles for epigenetic modifiers in brain disorders, as well as the development of new drugs to target these chromatin regulatory proteins in brain, will require a deeper understanding of how these proteins and their complex structures contribute to transcriptional mechanisms in the CNS in order to facilitate the design of small molecule inhibitors or agonists for therapeutic intervention. Current inhibitors do exist for lysine methylation *readers*, HATs, HMTs, and DNMTs,^237^^,^^238^ and the future identification of *readers* for novel histone modifications, such as histone monoaminylations, may also present novel opportunities for designing targeted strategies once their mechanisms of action and structures have been fully characterized. This can be aided by computational cheminformatics, utilizing available data on small molecule inhibitors to screen for chemical compounds that might disrupt specific histone modification *reader* interactions.^239^ Unfortunately, at this time, it is estimated that ∼90% of small molecule inhibitors cannot pass the BBB. Thus, additional strategies aimed at overcoming this hurdle, such as intranasal injections, vasoactive compounds and optimized nanoparticle delivery, will be critical for future treatments of brain disorders. Currently, various nanoparticles including liposomes, quantum dots, nanogels and gold nanoparticles, which can pass the BBB, have shown promise for the precise targeting and release of small molecular inhibitors for the treatment of certain neurological and other brain disorders (Figure 6A).^168^^,^^240^ Recently, BBB-crossing conjugates have been engineered which could in theory be used to deliver conjugated peptides and small molecules.^241^

### Utilizing growing molecular toolboxes to manipulate gene expression as a therapeutic strategy

As we have highlighted throughout this review, several neurological disorders have been shown to be caused by specific mutations in key epigenetic modifying genes. Thus, implementing CRISPR-Cas9-based genetic therapies represents a highly enticing avenue for the potential treatment of these disorders (Figures 6B–6D). These therapies might be able to be delivered intravenously or through the use of AAV viruses that are brain- (or even cell-type) specific.^242^^,^^243^ Additionally, the use of AAV viruses to overexpress wild-type mRNA could possibly be used to treat LoF mutations or correct a loss of proper copy number for epigenetic proteins (Figure 6F). While it may be many years before brain-specific CRISPR-based therapeutics will be realized to treat neurological disorders in humans, the development of such strategies is of particular interest for treating patients that have *de novo* mutations in epigenetic modifiers and could benefit from customized treatments. Recent innovations using Cas9 nickases, for example, allow for single base pair prime editing, which may also be ideal for correcting point-mutations that disrupt protein structure and/or function in brain disorders, as these approaches can be engineered to minimize off-target effects (Figure 6C).^244^^,^^245^ Additionally, dCas9 proteins can be targeted to select loci with a transcriptional activator or repressor, a strategy which could be utilized to regulate gene expression in the correct context (Figure 6D). dCas9 proteins have also been modified to utilize intein technologies to selectively deliver tethered small molecule inhibitors to specific loci of interest, which could represent a promising strategy in select cases.^246^ PROteolysis targeting chimeras (PROTACs) are another rapidly evolving technology that could be utilized in the treatment of neurological disorders.^247^ PROTACs use high affinity molecules to target specific proteins for degradation by endogenous ubiquitin pathways, historically used to target protein degradation in cancer (Figure 6E). Recently, PROTACs have been successfully designed to target neurological diseases, such as AD and Parkinson’s disease, where they can be designed to degrade Tau.^248^ Such PROTACs have been engineered to robustly cross the BBB and could be used to target epigenetic regulators in brain. This approach could be useful for the treatment of a multitude of brain disorders that result from the overexpression of epigenetic regulators (such as *BRWD1* in Down syndrome), or when there is a global imbalance of histone PTMs due to LoF mutations. An alternative strategy could also be to degrade a *reader* that is enhanced in its binding, or degrade a *writer* or *eraser* that is upregulated in response to an imbalance caused by a LoF mutation, as has previously been done in cancer models.^45^^,^^183^^,^^249^ Another potential therapeutic strategy utilizes antisense oligonucleotides (ASOs), which are short pieces of ssDNA/RNA molecules that can target mRNA for either degradation or alternative splicing.^168^^,^^240^ This strategy might be ideal for attempting to treat brain disorders that are caused by frameshift or truncating mutations and/or aberrant splicing, as they can induce exon skipping, which can then rescue protein expression with minimal LoF (Figure 6G). While these molecules do not naturally pass the BBB, they can be coupled to nanoparticles for delivery to achieve brain-specific targeting (Figure 6A).^168^^,^^240^ Altogether, the technologies available for treating brain disorders are rapidly evolving and, in the future, may be utilized to target epigenetic imbalances that precipitate disease. Again, understanding the precise biochemical mechanisms underlying these disease states will be vital for the design of these targeted therapeutic strategies, along with extensive testing in both cellular and *in vivo*.

## Conclusion: The future of neuroepigenetic therapeutics

Through the collective work of countless geneticists, chromatin biologists, chemical biologists, neuroscientists, and neuroepigenetics researchers, great strides have been made in the past two decades in the identification and characterization of genetic variants in epigenetic regulatory proteins and their contributions to brain disorders. Here, we have reviewed select critical genes and how they were identified, the brain-specific epigenetic processes that they affect and their contributions to disease. While the insights provided by these studies have been of great importance to the field, gaps in identifying genetic variants and then determining their molecular/biochemical mechanisms of action in brain remain. To close these gaps, we propose that integrating findings across multiple diverse disciplines using a combination of traditional and novel biochemical tools will be paramount. Such efforts promise to result in a more holistic understanding of how these genetic variants affect human health, and future exhaustive characterizations of these neuroepigenetic mechanisms will be necessary to inform targeted therapeutics in human patients.

## Acknowledgments

We would like to thank Dr. Jennifer Chan of the Maze Lab for her insights and edits during the review process. All Figures were created using BioRender. The authors would also like to acknowledge financial support for this manuscript from the 10.13039/100000025National Institute of Mental Health R01MH116900 (I.M.), 10.13039/100000071National Institute of Child Health and Human Development R01HD097088 (I.M.), 10.13039/100000026National Institute on Drug Abuse R01DA056595 (I.M.), and the 10.13039/100000011Howard Hughes Medical Institute (I.M.).

## Author contributions

Conceptualization: I.M., B.H.W., and N.I.A.; writing: B.H.W., N.I.A., and I.M.; review and editing: B.H.W., N.I.A., and I.M.; visualization: B.H.W. and N.I.A.

## Declaration of interests

The authors declare no competing interests.
